# Earlyto Mid-Term Results of Aortic Valve Neocuspidization for Rheumatic Aortic Valve Disease

**DOI:** 10.21470/1678-9741-2024-0412

**Published:** 2025-08-22

**Authors:** Mohamed Sanad, Mohamed Gabr, Ahmed ElDerie, Hatem Beshir, Mohamed Hegazy, Mohammed Abdallah, Sameh M. Said

**Affiliations:** 1 Department of Cardiothoracic Surgery, Faculty of Medicine, Mansoura University, Mansoura, Egypt; 2 Department of Cardiothoracic Surgery, Amreya General Hospital, Ministry of Health, Alexandria, Egypt; 3 Department of Anesthesiology and Surgical Critical Care, Faculty of Medicine, Mansoura University, Mansoura, Egypt; 4 Department of Cardiology, Faculty of Medicine, Mansoura University, Mansoura, Egypt; 5 Department of Surgery, Division of Pediatric and Adult Congenital Cardiac Surgery, Maria Fareri Children’s Hospital, Westchester Medical Center, New York Medical College, Valhalla, New York, United States of America; 6 Department of Cardiothoracic Surgery, Faculty of Medicine, Alexandria University, Alexandria, Egypt

**Keywords:** Aortic Valve Insufficiency, Reoperation, Rheumatic Heart Disease, Cardiopulmonary Bypass, Pathologic Constriction, Coronavirus, Patient Discharge.

## Abstract

**Introduction:**

Recently, there has been a widespread use of aortic valve neocuspidization, but there is limited data regarding rheumatic heart disease. In this study, we reviewed our experience.

**Methods:**

A total of 33 patients (22 men, 66.7%) with rheumatic aortic valve disease (mean age 39.36 ± 10.65 years) underwent aortic valve replacement between June 2019 and October 2023.

**Results:**

The most common pathology was severe stenosis (14 patients, 42.4%), with bicuspid morphology in 11 patients (33.3%). The mean cardiopulmonary bypass and aortic cross-clamping times were 151 ± 24.26 and 127 ± 21.05 minutes, respectively. There was no perioperative mortality. One patient who developed significant aortic regurgitation underwent valve replacement prior to discharge. The pre-discharge average peak/mean gradients were 12 ± 3.7/6 ± 2 mmHg, respectively. Follow-up was complete (mean: 31.54 ± 12.94 months). There were two late mortalities (6%), one due to endocarditis and another due to coronavirus disease. One patient (3%) needed a permanent pacemaker one year later. Overall survival at one, two, and four years were 97%, 97%, and 94% respectively, and freedom from reoperation was consistent at 97%. The peak/mean gradients remained low at one and three years (12 ± 2.7 mmHg/4.8 ± 1.7 mmHg and 10.14 ± 4.02/4.4 ± 2.3 mmHg, respectively). Overall four-year freedom from at least moderate regurgitation was 97%.

**Conclusion:**

Our data shows promising results for this procedure in rheumatic pathology. The hemodynamic data is satisfactory and the earlyto mid-term results are encouraging; however, long-term data is needed to determine durability.

## INTRODUCTION

**Table:** 

Abbreviations, Acronyms & Symbols				
ANOVA	= Analysis of variance		IHD	= Ischemic heart disease
AR	= Aortic regurgitation		IRB	= Institutional Review Board
AS	= Aortic stenosis		LV	= Left ventricular
AV	= Aortic valve		LVEF	= Left ventricular ejection fraction
AVA	= Aortic valve area		LVMi	= Left ventricular mass index
AVNeo	= Aortic valve neocuspidization		MG	= Mean gradient
AVR	= Aortic valve replacement		MS	= Mean squares
AXC	= Aortic cross-clamping		NYHA	= New York Heart Association
BAV	= Bicuspid aortic valve		PG	= Peak gradient
BSA	= Body surface area		PPM	= Permanent pacemaker
COPD	= Chronic obstructive pulmonary disease		RHD	= Rheumatic heart disease
CPB	= Cardiopulmonary bypass		SD	= Standard deviation
df	= Degrees of freedom		SS	= Sum-of-squares
DM	= Diabetes mellitus		SVD	= Structural valve deterioration
EDD	= End-diastolic diameter		TAV	= Tricuspid aortic valve
ESD	= End-systolic diameter		TEE	= Transesophageal echocardiography
HT_N_	= Hypertension		TTE	= Transthoracic echocardiogram

Autologous pericardium has been long used for aortic valve (AV) repair^[^1^]^ and/or replacement^[^2^]^ with an average of 63% freedom from reoperation at 48 months as reported in a study of 87 patients by Gross et al.^[^3^]^. Due to this limited durability, the utilization of autologous pericardium as a leaflet/valve substitute has fallen out of favor.

Ozaki et al. have recently reported the utilization of glutaraldehyde-treated autologous pericardium for replacement of the non-repairable AV with encouraging results^[^4^]^. Their technique, however, is based on Duran’s technique^[^5^]^ but they created a more standardized way of reconstruction of the AV leaflets which could be in part responsible for the better results reported. With these encouraging results, the Ozaki procedure, or the AV neocuspidization (AVNeo), has been widely expanding in many centers worldwide and extended to both the pediatric^[^6^]^ and adult population^[^7^]^ with good earlyand mid-term results. The procedure expanded to include almost all AV pathologies, whether congenital or acquired.

Most of the reported experience in AVNeo involves patients with degenerative valve disease, with limited information on the behavior of the autologous pericardium in the setting of rheumatic heart disease (RHD). RHD continues to be the predominant pathology in many low-to-middle income countries, especially in children and young adults^[^8^]^. In this population and in these countries, the choice of the prosthesis is challenging, and AVNeo may represent a reasonable alternative; however, no data is available on the results of AVNeo in this particular group of patients.

In the current study, we present our earlyto mid-term results of AVNeo in patients with rheumatic AV disease.

## METHODS

The Institutional Review Board (IRB) approved the current study (IRB 23.11.2623) on May 12, 2023. An individual patient’s consent was waived by the IRB due to the retrospective nature of the study and the minimal-to-no risk involved in chart reviews. Data were collected from clinical records, operative reports, and follow-up clinic visits.

### Patients’ Characteristics (Table 1)

The current study includes a total of 33 adult patients (22 men, 66.7%) with isolated rheumatic AV disease. Their mean age is 39.36 ± 10.65 years old. The patients made the decision to choose the AVNeo procedure after full discussion of all pros and cons of the procedure as well as other available AV replacement (AVR) options such as mechanical and biological prostheses.

**Table 1:** Patients’ characteristics.

Variable	Number	Frequency (%)
Demographics		
Men	22	66.7
Age (years) (mean ± SD)	39.36 ± 10.65	
20 - 30 years	9	27.3
31 - 40 years	8	24.2
41 - 50 years	10	30.3
51 - 60 years	6	18.2
BSA (m^2^) (mean ± SD)	1.84 ± 0.138	
NYHA class		
III	24	72.7
IV	1	3
Comorbidities		
HT_N_	8	24.2
DM	3	9.1
COPD	2	6.1
IHD	1	3
Aortic valve		
Pathology		
AR	14	42.4
AS	12	36.4
Mixed AS/AR	7	21.2
Morphology		
TAV	22	66.7
BAV	11	33.3
Type 1	1	30.3
Type 0	10	3

AR=aortic regurgitation; AS=aortic stenosis; BAV=bicuspid aortic valve; BSA=body surface area; COPD=chronic obstructive pulmonary disease; DM=diabetes mellitus; HTN=hypertension; IHD=ischemic heart disease; NYHA=New York Heart Association; SD=standard deviation; TAV=tricuspid aortic valve

### Operative Technique

**Fig. 1:**
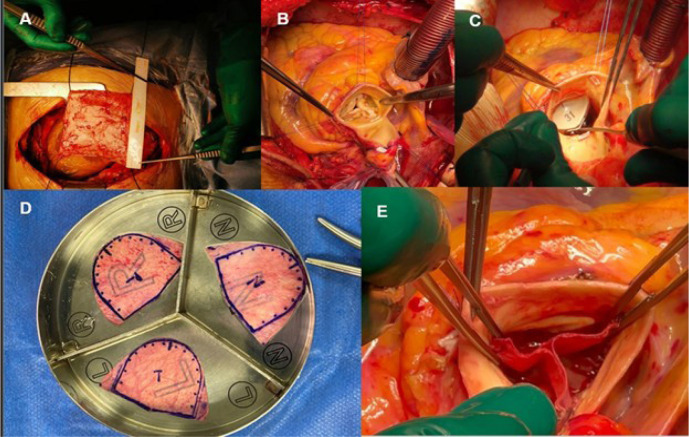
A-E) Intraoperative photos showing the technique of aortic valve neocuspidization using autologous pericardium. (A) After median sternotomy, a large sheet of the anterior pericardium is harvested and treated with glutaraldehyde 0.6% for 10 minutes; (B) transverse aortotomy with/without aortic transection showing a heavily calcified tricuspid aortic valve; (C) once the aortic annulus is debrided, the neo-aortic cusps are being sized using standard sizer; (D) all patients had three equally created leaflets using the provided template; (E) the final appearance of a tricuspid neo-aortic valve is shown.

The technical aspects of the AVNeo procedure have been previously published, and we followed the same operative steps in the current study. Briefly, after median sternotomy, a large sheet of the anterior pericardium was harvested and treated with glutaraldehyde 0.6% for 10 minutes (Figure 1A), followed by saline wash three times, each for six minutes. Once cardiopulmonary bypass (CPB) is initiated and cardioplegic arrest is achieved, the AV is exposed through a transverse aortotomy (Figure 1B). The AV leaflets are resected, and the annulus is debrided thoroughly, followed by sizing the AVNeo leaflets (Figure 1C). We used three equal size leaflets (Figure 1D) that are fashioned according to the provided template. While it is not infrequent that all sinuses are not of equal sizes, we always were able to create three equal size leaflets as described by Ozaki et al. in their modification of the original technique. This includes cases of bicuspid AV as well. This often requires changing or altering the location of the native commissure or creating a new location for it. The new leaflets are sewn in with three running polypropylene sutures, and new commissures are created. The final result was a tricuspid valve morphology (Figure 1E) (Video 1). This was followed by aortotomy closure and completion of the procedure in the standard fashion.

**Video 1:**
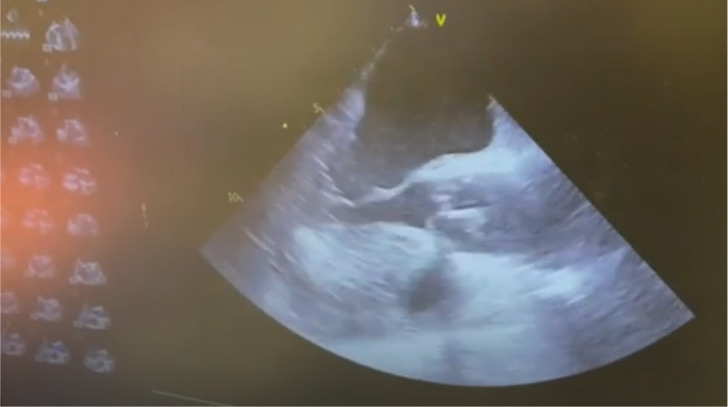
Final appearance of the reconstructed aortic valve on transesophageal echocardiogram post bypass. Notice the large coaptation surface. Link: https://youtu.be/PBhRNhhW5bE

### Statistical Analysis

Baselines characteristics are reported as mean ± standard deviation, median and interquartile range, or ranges for continuous variables, and as counts and percentages for categorical variables. Kaplan-Meier curves are generated to provide freedom from reoperation and significant aortic regurgitation (AR).

## RESULTS

### Patients’ Baseline Characteristics

The study included 22 men (66.7%) and 11 women (33.3%) with a mean body surface area of 1.84 ± 0.14 m^2^. All patients had a highly suggestive or confirmatory history of rheumatic fever which was validated by communication with their referring cardiologist, previous medical records, or previous long-term treatment with penicillin injections. This was confirmed later with pathological examination of the resected AV cusps. The mean European System for Cardiac Operative Risk Evaluation II was 0.96 ± 0.17%. Most of the patients (24, 72.7%) were in New York Heart Association (NYHA) class III, while one (3%) was in NYHA class IV. Comorbidities included hypertension in eight patients (24.2%), diabetes mellitus in three (9.1%), chronic obstructive pulmonary disease in two (6.1%), and ischemic heart disease in one (3%). All operations were primary cardiac procedures. The most common indication for surgery was isolated severe aortic stenosis (AS) (14 patients, 42.4%), followed by isolated severe AR (36.4%) (Table 1).

### Anatomical Characteristics and Concomitant Pathology

Most of the patients had tricuspid AV morphology (22 patients, 66.7%). The mean aortic annulus was 23.091 ± 2.81 mm. The mean AV area (AVA) was 1.55 ± 1.04 mm^2^, and the mean peak gradient (PG) and mean gradient (MG) were 63 ± 38 and 38.3 ± 25.1 mmHg, respectively (Table 2).

**Table 2:** Echocardiographic data (mean and standard deviation).

Variable	Preoperative	Post-bypass TEE	Pre-discharge	1 month	1 year	3 years	ANOVA
AVA (mm^2^)	1.55 ± 1.04	2.71 ± 0.6	2.64 ± 0.45	2.68 ± 0.45	2.55 ± 0.35	2.6 ± 0.29	SS = 9.876
							MS = 6.53
							∑ = 0.303
							df = 1.513
							F = 10.89
							*P* = 0.002
PG (mmHg)	63 ± 38	15 ± 4.9	12 ± 3.7	12 ± 4.2	12 ± 2.7	10.14 ± 4.02	SS = 28369.8
							MS = 26045.6
							∑ = 0.218
							df = 1.089
							F = 29.55
							*P* < 0.0001
MG (mmHg)	38.3 ± 25.1	6.8 ± 3.4	5.5 ± 2.3	5.7 ± 2.5	4.8 ± 1.7	4.36 ± 2.35	SS = 9.876
							MS = 6.53
							∑ = 0.213
							df = 1.065
							F = 25.64
							*P* = 0.02
EDD	53.24 ± 8.87	50.93 ± 9.71	50.26 ± 6.8	49.52 ± 5.47	46.52 ± 4.98	44.43 ± 3.78	SS = 831.82
							MS = 166.36
							∑ = 0.28
							df = 1.42
							F = 5.462
							*P* = 0.021
ESD	34.7 ± 7.02	33.81 ± 5.44	34.12 ± 6.61	34.86 ± 6.45	33.13 ± 5.69	29.93 ± 4.41	SS = 246.714
							MS = 129.67
							∑ = 0.38
							df = 1.9
							F = 3.59
							*P* = 0.045
LVEF (%)	62.12 ± 8.71	65.54 ± 8.91	62.13 ± 9.58	61.26 ± 8.99	61.09 ± 7.24	62.93 ± 6.33	SS = 611.8
							MS = 316.22
							∑ = 0.38
							df = 1.94
							F = 4.44
							*P* = 0.078
Left ventricular mass	272.29 ± 61.85		254.74 ± 62.03	238.66 ± 66.13	170.7 ± 39.22	140.69 ± 28.4	SS = 149610.8
							MS = 70326.3
							∑ = 0.53
							df = 2.13
							F = 17.14
							*P* = 0.007
LVMi (g/m^2^)	148.39 ± 31.93		138.43 ± 33.28	131.8 ± 36.83	90.63 ± 16.92	75.09 ± 12.97	SS = 45132.06
							MS = 21451.71
							∑ = 0.53
							df = 2.13
							F = 16.16
							*P* = 0.002
Post-AVNeo AR degree							
None			19	22	25	31	
Trivial			13	10	5	0	
Mild			0	0	2	0	
≥ Moderate			1^*^	0	0	1^**^	

* Underwent standard aortic valve replacement with a mechanical prosthesis prior to discharge

** One patient progressed from mild to moderate AR and has been followed closely with no planned intervention currently

A repeated measures ANOVA was done between each continuous variable at different instances of follow-up. Mauchly’s test indicated that the assumption of sphericity had been met or violated. Greenhouse-Geisser correction was used to determine that mean parameter differed statistically between time points. Post-hoc analysis with a Bonferroni adjustment was done. Tests of Within-Subjects Effects Pairwise comparisons were done. *P*-values of significance were calculated for the Greenhouse-Geisser if the data violates the assumption of sphericity and the epsilon (ɛ) for Greenhouse-Geisser in the Mauchly’s Test of Sphericity table was < 0.75, while *P*-values for Huynh-Feldt were calculated if the data violated the assumption of sphericity and the epsilon (ɛ) for Greenhouse-Geisser in the Mauchly’s Test of Sphericity table is > 0.75

ANOVA=analysis of variance; AR=aortic regurgitation; AVA=aortic valve area; AVNeo=aortic valve neocuspidization; df=degrees of freedom; EDD=end-diastolic diameter; ESD=end-systolic diameter; LVEF=left ventricular ejection fraction; LVMi=left ventricular mass index; MG=mean gradient; MS=mean squares; PG=peak gradient; SS=sum-of-squares; TEE=transesophageal echocardiography

One patient (3%) had concomitant high-grade stenosis of the left anterior descending coronary artery and underwent coronary artery bypass grafting with the left internal mammary artery (Table 1).

### Operative Data and Early results

The mean CPB and aortic cross-clamping (AXC) times were 151 ± 24.26 and 127 ± 21.05 minutes, respectively. Isolated AVR was the main procedure (32 patients, 97%). All patients had three equally designed AVNeo leaflets. The mean right-, left-, and non-coronary leaflets sizes were 26.21 ± 3.39 mm, 25.67 ± 3.15 mm, and 26.27 ± 3.19 mm, respectively. The minimum leaflet size was 21 mm, while the largest was 33 mm. There was a significant drop in the PG (15 ± 4.9) and MG (6.8 ± 3.4) from baseline (*P* < 0.0001 and *P* = 0.02, respectively). The preoperative mean left ventricular mass index (LVMi) was 148.39 ± 31.93 g/m^2^, which also changed significantly postoperatively (138.43 ± 33.28; *P* = 0.002) (Table 2).

Post-bypass transesophageal echocardiography (TEE) showed competent AVNeo in 23 patients (69.7%), trivial AR in nine (27.3%), and mild AR in one (3%). The mean AVA was 2.71 ± 0.60 mm2, which was significant compared to baseline (*P* = 0.002). There was no perioperative mortality or conversion to standard prosthetic AVR. There was no stroke, and there were two early reoperations: one patient (3%) required re-exploration for bleeding, and another patient (3%) developed neo-right coronary cusp failure with resulting severe AR one week after the initial procedure, which was attributed to suture line dehiscence. This patient underwent mechanical AVR prior to hospital discharge. The mean ventilation time was 4.88 ± 2.15 hours, while the mean intensive care unit and hospital stays were 2.28 ± 1.17 days and 4.97 ± 1.89 days, respectively. The pre-discharge mean AVA was 2.64 ± 0.45 mm^2^, while the pre-discharge average PG and MG were 12 ± 3.7 mmHg and 6 ± 2 mmHg, respectively.

### Follow-up

Follow-up was complete with a mean of 31.54 ± 12.94 months. In addition to intraoperative TEE, all patients received pre-discharge transthoracic echocardiogram (TTE) and were followed in the outpatient clinic by both clinical evaluation and TTE at one, three, six, and 12 months and yearly thereafter.

### Late Results

**Fig. 2:**
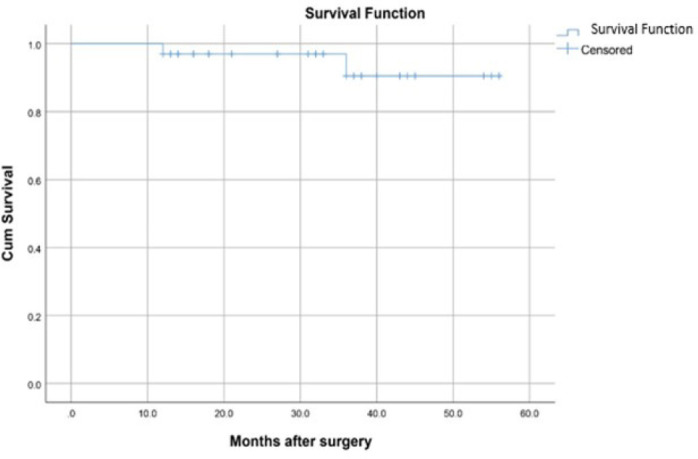
Kaplan-Meier curve for survival in the current study.

**Fig. 3:**
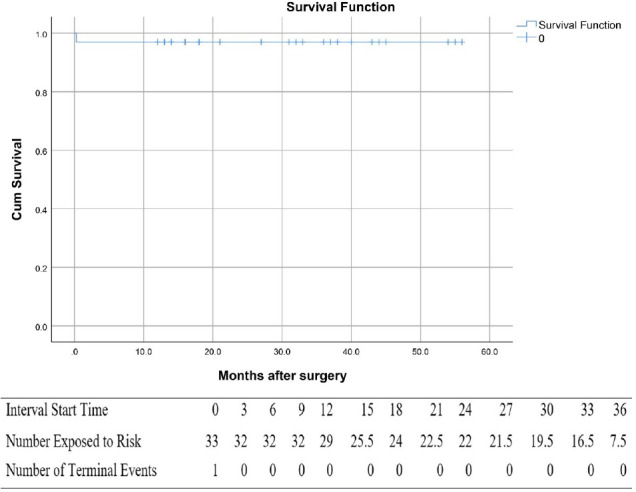
Kaplan-Meier curve showing freedom from aortic valve reoperation.

There were two late mortalities (6%). One patient developed infective endocarditis (3%) with aortic root abscess three years after the primary procedure, and a second one (3%) died of non-cardiac related cause (coronavirus disease with multiorgan failure 17 days after the operation). One patient (3%) required permanent pacemaker (PPM). This was a 42-year-old man with a heavily calcified bicuspid AV and chronic liver disease. He developed complete heart block a year after his uneventful AVNeo procedure and underwent PPM placement. There was no clear reason for this to happen a year after his valve procedure. Overall survival at one, two, and four years were 97%, 97%, and 94%, respectively (Figure 2). Freedom from cardiac-related deaths were 100%, 100%, and 97% at one, two, and four years, respectively. Freedom from AV reoperation at one, two, and four years was consistent at 97% (Figure 3).

### Echocardiographic Follow-up (Table 2)

**Fig. 4:**
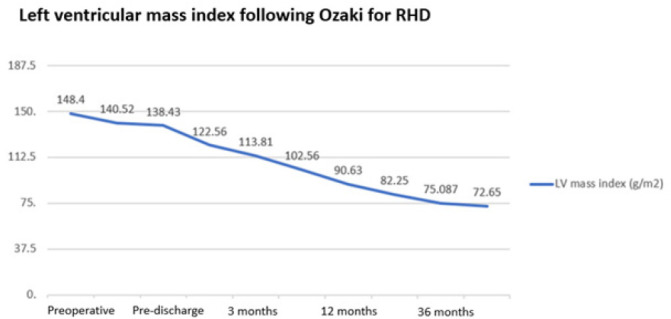
The continued regression of the left ventricular (LV) mass index is shown, which was consistent during the follow-up period. RHD=rheumatic heart disease.

**Fig. 5:**
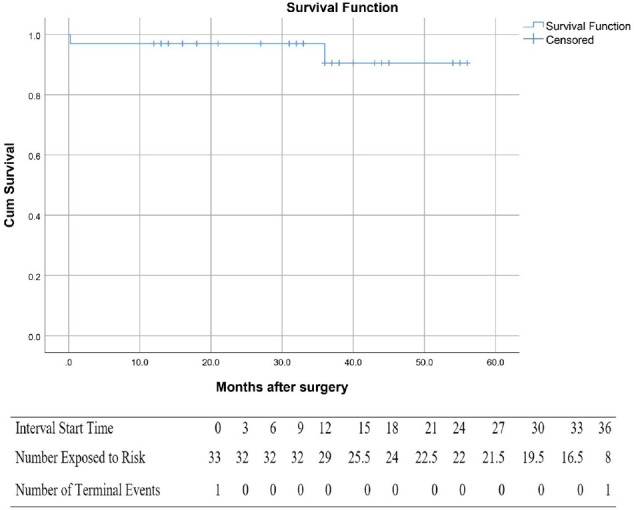
Kaplan-Meier curve showing freedom from moderate or more aortic regurgitation during the follow-up period.

At one-month follow-up, the mean AVA was 2.68 ± 0.45 mm^2^, and the average PG and MG were 12 ± 4.2 mmHg and 5.7 ± 2.5 mmHg, respectively. These excellent hemodynamics continued during the follow-up period with average AVA at oneand three-year follow-up of 2.55 ± 0.35 mm^2^ and 2.6 ± 0.29 mm^2^, respectively. The average PG/MG remained low as well with oneand three-year follow-up of 12 ± 2.7 mmHg/4.8 ± 1.7 mmHg and 10.14 ± 4.02/4.4 ± 2.3 mmHg, respectively (Table 2). The LVMi continued to regress during the follow-up period with a mean LVMi at one month of 131.84 ± 35.90 g/m^2^, which was almost normalized at the one-year follow-up (90.63 ± 16.38g/m2) (Figure 4). At one and three months postoperatively, no AR was detected in the majority of patients (24, 75%), while trivial AR was present in eight (25%). No patient had mild or more AR, which was consistent at the six-month follow-up. Two patients (6.25%) developed mild AR in one year. At three-year follow-up, one patient (6.67%) progressed from mild to moderate AR and remained stable. Bicuspid AV was not an independent risk factor for AR (*P* = 0.91). Overall freedom from moderate or more AR at four years was 97% (Figure 5).

## DISCUSSION

The AVNeo program at our institution was established in June 2019. A total of 86 AVNeo cases were performed during the current study time interval (from June 2019 to October 2023) and included the 33 patients with isolated rheumatic AV disease that constituted the current study cohort.

RHD continues to be the main etiology of valve disease in low-income countries; most of the patients are among the young age group category, but with advanced valve pathology that is mostly irreparable. This cohort is unique, and this younger age represents a challenge in selecting the most suitable prosthesis. Long-term data of bioprostheses in young patients are unfavorable with rapid early deterioration and limited durability, which subjects many of these young patients to repeat operations/interventions, escalating their procedural risks and lowering their long-term survival. Freedom from structural valve deterioration (SVD) at 10, 15, and 20 years was 94.2%, 78.6%, and 48.5%, respectively, in the study by Bourguignon et al.^[^9^]^. This drops even further in patients < 60 years of age according to the same study. The same data was found in the study by David et al., where they reported 20-year freedom from SVD in > 1,000 patients to be 63.4% and 29.2% for the entire cohort and for those < 60 years of age, respectively^[^10^]^. Mechanical prostheses, while durable and associated with better survival compared to bioprostheses^[^11^]^, are difficult to manage due to the needed life-long anticoagulation which represents a major disadvantage in these younger patients who are seeking an active lifestyle. The long-term survival advantage of mechanical prostheses appears to be more prominent in the younger age group according to the study by Goldstone et al. where the authors showed a significantly higher 15-year mortality in those between 45 and 54 years of age^[^12^]^.

Ross procedure remains the best option for young patients and has been associated with survival that is close to and/or matches normal population according to several recent studies^[^13^,^14^]^. However, the Ross procedure is more complex, not easily reproducible, and the limited availability of homografts outside the United States of America makes this an unrealistic option.

Ozaki et al. standardized the technique of reconstruction of the AV with the autologous pericardium via specially designed templates. This standardization is what makes the procedure reproducible and simplifies its steps. It has several advantages in this particular age group. Absence of stent in the AVNeo creates a low/insignificant left ventricular (LV) outflow tract gradient which translates into better hemodynamics compared to standard prostheses^[^15^]^. The average PG was 23.1 ± 14.5 mmHg and 19 ± 8.6 in one week and 26 months, respectively, in the study by Iida et al.^[^16^]^. In the current study, the drop in the average PG and MG was significant immediately postoperatively (63 ± 38 to 15 ± 4.9 mmHg [*P* < 0.0001] for the PG and 38.3 ± 25.1 to 6.8 ± 3.4 mmHg [*P* = 0.02] for the MG). This low gradient was maintained during the follow-up period with a three-year follow-up PG and MG of 10.14 ± 4.02 and 4.36 ± 2.35 mmHg, respectively (Table 2). This did not only translate to excellent hemodynamics, but we observed continuous regression of the LV mass, which started at discharge and persisted during follow-up and up to three years. The baseline LVMi was 148.39 ± 31.93 g/m^2^, which regressed significantly during the follow-up period (75.09 ± 12.97 g/m^2^ at three-year follow-up; *P* = 0.002). This was observed in those with isolated AS and isolated AR as well (Table 3), and these data are consistent with others. Sharma et al.^[^17^]^ reported that there was a 25% reduction in LVMi in six months and 30% in 7-24 months, which is consistent with our data. The possible explanation is the low gradient related to the AVNeo procedure, which is in contrast to bioprosthetic and mechanical prostheses but close, if not similar, to the Ross and homograft data. We believe the low gradient is what allows the myocardial recovery and the LV mass regression.

**Table 3:** Mean and standard deviation for the baseline and changes in the left ventricular mass index (LVMi) between those with isolated aortic stenosis (AS) and those with isolated aortic regurgitation (AR) at baseline, postoperatively, and during the oneand three-year follow-up.

LVMi	Severe AR	Severe AS	Sig.
Preoperative LVMi	155.88 ± 35.75	148.68 ± 36.61	0.59
Pre-discharge LVMi	139.46 ± 37.27	146.16 ± 37.96	0.58
1-month LVMi	127.49 ± 36.19	130.87 ± 40.61	0.61
12-month LVMi	96.66 ± 18.49	90.16 ± 16.15	0.38
36-month LVMi	80.26 ± 12.68	75.091 ± 12.97	0.38

The reported average PG and MG were 16.1 ± 8.1 mmHg and 8.9 ± 3.8 mmHg, respectively, by Krane et al. and remained stable at one-year follow-up^[^18^]^. In another recent Vietnamese study, the average PG and MG were 11.9 ± 2.3 mmHg and 6.8 ± 1.4 mmHg, respectively, at one week^[^19^]^. The low gradient may be the result of combined absence of the stent and the larger effective orifice area of the AVNeo^[^20^]^. In our study, the mean AVA increased significantly compared to baseline (1.55 ± 1.04 to 2.71 ± 0.6 immediately postoperatively; *P* = 0.002). This increase remained during the follow-up period. This low gradient may be a key factor in determining the long-term durability of the AVNeo especially in younger patients and those with small aortic annulus where there is a higher risk of patient-prosthesis mismatch with standard aortic prosthetic replacement. Other potential advantages include the lack of need for life-long anticoagulation, which makes the AVNeo desirable in those who want to continue an active lifestyle, or in women in their child-bearing period, in addition to retaining the possibility of performing AVR with all other options if reoperation is required.

The literature was enriched recently with several studies on the utilization and results of the Ozaki AV reconstruction in both children and adults. This comes in handy with the current widespread application of the procedure. No long-term data is available yet for the AVNeo procedure to compare with other AVR options, but the earlyand mid-term results are encouraging. The indications for AVNeo procedure have been expanded to include all isolated AV pathologies. There is debate in regard to performing the procedure in extensive cases of endocarditis affecting the aortic annulus with/without root abscess and those requiring concomitant aortic root replacement. This is based on the need for more complex patching of the aortic root and or the need for simultaneous graft replacement which may result in suboptimal hemodynamics with subsequent lower durability due to the theoretical loss of native annulus and aortic wall^[^21^]^.

While the AVNeo as a procedure is simple compared to the Ross procedure or the mere homograft replacement of the aortic root, it is associated with relatively longer CPB and AXC times in comparison with standard prosthetic AVR. This has been a factor in increased perioperative morbidities, with 1.4% increased risk for every minute increased in these times, according to Ranucci et al.^[^22^]^. In the current study, the mean CPB and AXC times were 151 ± 24.26 and 127 ± 21.05 minutes, respectively. This has been in line with the literature, and in fact shorter than some studies.

The need for PPM after AVNeo has been quite low. The suture line is away from the conduction tissue and unless the injury to the conduction tissues results from extensive debridement in heavily calcified AV, the incidence should remain theoretically low, which gives this procedure another advantage. In the current study, only one patient needed late PPM placement, which was one year after the initial procedure. This is also in line with the literature. In the initial Ozaki data, the authors reported one case of PPM. Koechlin et al. reported no cases of PPM after isolated AVNeo^[^23^]^, while it was 8.6% for concomitant AVNeo cases.

While AVNeo has been performed in all AV pathologies, the outcomes in certain pathologies remain unknown and whether using the patients’ own pericardium for AV reconstruction in these cases should be considered is uncertain. The pericardium is affected in RHD as part of the initial pancarditis phase that occurs. This may change the nature of the pericardium and renders it unsuitable for the AVNeo, however we did not encounter that in any patient in the current series. The long-term durability, however, remains to be determined. There is another concern related to the risk of recurrence of rheumatic fever, which is higher in younger patients and may result in recurrent valvular pathology in the newly constructed valve^[^24^]^. The literature is very scant when it comes to the outcomes of AVNeo in RHD, which makes our series very unique.

The RHD burden cannot be ignored with reported direct costs to healthcare of 11.8 billion dollars in India and 50 million in Uganda^[^25^]^. This disease continues to be the most common cause of valve degeneration in Egypt especially in the younger age group. With the current challenges in healthcare and limited resources, the Ozaki AVNeo procedure represents a great cost-effective alternative for AVR in these countries in addition to the previously mentioned advantages.

### Limitations

The current study is limited by its small number of patients, but it constitutes a relatively large number for this unique pathology. Longer-term follow-up will be needed to determine the long-term durability of the AVNeo in this particular group of patients.

## CONCLUSION

AVNeo is a reproducible technique with a very short learning curve. Our data shows promising results for this procedure in those with rheumatic valve pathology. The hemodynamic data is satisfactory, and the earlyto mid-term results are encouraging. The lack of anticoagulation makes it a very desirable option for AVR in young patients. Long-term data is needed to determine durability.
